# Acute Hypoxic Respiratory Failure Secondary to an Intermittent Interatrial Shunt

**DOI:** 10.7759/cureus.85518

**Published:** 2025-06-07

**Authors:** Angela Baquiran, Yongdeok B Shin, Sridhar Neralla

**Affiliations:** 1 Internal Medicine, Mary Washington Healthcare, Fredericksburg, USA; 2 Critical Care Medicine, Mary Washington Healthcare, Fredericksburg, USA

**Keywords:** asd (atrial septal defect), hypoxia, intermittent right-to-left shunting, transesophageal echocardiography (tee), valsalva maneuver

## Abstract

Intermittent right-to-left shunting through an atrial septal defect (ASD) is a rare but significant cause of acute hypoxic respiratory failure. This case highlights the diagnostic and management challenges associated with an intermittent interatrial shunt. A 65-year-old man presented with profound hypoxia requiring mechanical ventilation. Despite an extensive initial workup, including a computed tomography angiogram and transthoracic echocardiography with a bubble study, which was negative for an intracardiac shunt, no definitive cause for his hypoxia was identified. Given persistent clinical suspicion and the episodic nature of the isolated hypoxia, a transesophageal echocardiogram was performed, revealing a 2.6 cm² secundum ASD with intermittent right-to-left shunting, predominantly occurring during maneuvers that increased intrathoracic pressure, such as Valsalva. Key differentials, including pulmonary embolism and pneumonia, were systematically excluded. The patient underwent successful percutaneous ASD closure, leading to significant improvement in oxygenation, with follow-up demonstrating sustained normoxia and stable oxygen saturation. This case underscores the importance of considering an intracardiac shunt in patients with unexplained, episodic hypoxia and demonstrates the necessity of advanced imaging modalities for accurate diagnosis.

## Introduction

Atrial septal defects (ASDs) are among the most common congenital cardiac anomalies, with an estimated prevalence of 1 to 2 per 1,000 live births [1]. They arise from an incomplete closure of the atrial septum during fetal development and persist into adulthood when spontaneous closure does not occur. The natural history of ASDs varies widely, depending on the size of the defect and the resultant hemodynamic burden on the heart and pulmonary circulation.

Most ASDs result in a left-to-right shunt due to the pressure gradient between the left and right atria, leading to increased pulmonary blood flow and volume overload of the right heart chambers. Over time, chronic right-sided volume overload can lead to pulmonary hypertension, atrial arrhythmias, and right heart failure [2]. However, under certain pathophysiologic conditions, including elevated right atrial pressure, pulmonary hypertension, or transient hemodynamic shifts, the shunt may reverse, resulting in right-to-left blood flow and subsequent systemic hypoxia [3].

Intermittent right-to-left shunting through an ASD is a rare and underreported phenomenon, as it can be transient and challenging to diagnose. Unlike persistent right-to-left shunting seen in conditions such as Eisenmenger syndrome, intermittent shunting occurs sporadically due to physiological changes, including variations in intrathoracic pressure, Valsalva maneuvers, or acute increases in right atrial pressure from pulmonary embolism or tricuspid regurgitation [4]. Several anatomical factors, such as aortic root dilation, the presence of a persistent Eustachian valve, or an atrial septal aneurysm, may predispose patients to intermittent shunting by dynamically altering atrial pressures and flow patterns. Due to its episodic nature, intermittent shunting is often overlooked, leading to delayed diagnosis and potential mismanagement, including prolonged hypoxia or inadequate treatment.

Patients may present with paradoxical embolism, cryptogenic stroke, transient ischemic attacks, or severe hypoxia, particularly when in an upright position due to platypnea-orthodeoxia syndrome [5]. Detecting intermittent right-to-left shunting poses significant challenges, as standard imaging modalities, including transthoracic echocardiography (TTE) with bubble contrast, may fail to capture transient episodes of shunting. Transesophageal echocardiography (TEE), especially when combined with provocative maneuvers such as Valsalva, remains the gold standard for diagnosing intermittent shunting [6]. When TEE is inconclusive or not feasible, transcranial Doppler (TCD) with bubble study may be utilized to detect functional shunting, given its high sensitivity (97%) and specificity (93%) for identifying paradoxical embolism and microembolic signals [7].

The management of ASD-related right-to-left shunting depends on the severity of symptoms and the underlying hemodynamic status. While small, asymptomatic ASDs may not require intervention, symptomatic patients with significant right-to-left shunting are typically candidates for percutaneous or surgical closure of the defect. Percutaneous ASD closure is the preferred approach in suitable cases, as it effectively prevents further shunting, reduces the risk of embolic events, and improves oxygenation with minimal procedural morbidity. However, in cases where anatomical constraints preclude percutaneous closure, surgical repair remains a viable alternative [8].

This case underscores the importance of maintaining a high index of suspicion for intracardiac shunting in patients with unexplained hypoxia. It also highlights the diagnostic utility of TEE and the role of percutaneous closure in managing symptomatic right-to-left shunting. Early recognition and intervention are crucial to preventing long-term complications such as paradoxical embolism, stroke, and progressive right heart dysfunction.

## Case presentation

Clinical history and presentation

In July 2024, a 65-year-old man with a past medical history of hypertension, hyperlipidemia, pituitary tumor resection (with resulting adrenal insufficiency and hypothyroidism), and obesity (BMI 40.65 kg/m²) presented to the emergency department with acute-onset dyspnea and severe hypoxemia. His wife reported that he had experienced several days of gastrointestinal symptoms, including profuse vomiting and watery diarrhea, which had been progressively improving until a rapid and unexpected worsening of respiratory status occurred. EMS recorded an O₂ saturation in the 40s. In the ED, he remained hypoxic despite BiPAP and was emergently intubated.

Initial ABG on BiPAP showed PaO₂ 32.6 mmHg, pCO₂ 27.3 mmHg, and pH 7.3, indicating severe hypoxemic respiratory failure without hypercapnia or acidemia. CTA of the chest excluded pulmonary embolism. Chest imaging showed only mild atelectasis and no signs of consolidation, pulmonary edema, or infiltrates. Laboratory workup revealed lactic acidosis (lactate 6.0 mmol/L; reference range: 0.70 - 2.10 mmol/L) that improved with supportive care, and a viral respiratory panel was positive for parainfluenza virus. Given the absence of clear cardiopulmonary pathology, initial hypoxemia was attributed to viral pneumonia with possible aspiration, supported by a history of forceful vomiting.

The patient responded quickly to supportive treatment, was extubated the following morning, and transferred to the floor from the ICU.

Hospital course and recurrence of hypoxemia

On hospital day 5 (four days post-extubation), the patient developed progressive abdominal distention, nausea, and recurrence of severe hypoxemia. Repeat ABG showed PaO₂ 33.3 mmHg and A-a gradient of 77.2 mmHg. Imaging (X-ray and CT abdomen) revealed diffuse small bowel dilation consistent with ileus, without obstruction or transition point. The patient had experienced persistent ileus for approximately 4-5 days by this point, with decreasing bowel sounds, gaseous distention, and minimal bowel movements despite diarrhea early in the course. The ileus was attributed to viral gastroenteritis and possibly unrecognized pancreatitis (lipase 537 U/L; reference range: 23 - 300 U/L).

A nasogastric (NG) tube was placed and yielded over 2 liters of bilious emesis. Notably, following this decompression event, the patient's oxygenation rapidly normalized: PaO₂ increased to 253.2 mmHg on the same FiO₂ and A-a gradient improved dramatically to 8.3 mmHg (Table 1). This marked improvement was temporally correlated with NG decompression and spontaneous emesis.

**Table 1:** Arterial blood gas changes pre- and post-emesis Arterial blood gas (ABG) values before and after emesis demonstrating significant changes in oxygenation parameters. The alveolar-arterial (A–a) oxygen gradient, elevated prior to emesis, normalized afterward, indicating improved gas exchange. PaO₂: partial pressure of arterial oxygen.

Timepoint	Laboratory Parameter	Measured Value	Interpretation	Reference Range
Pre-emesis	PaO₂	33.30%	Low	92–98.5%
Pre-emesis	A–a Gradient	77.2 mmHg	High	5–10 mmHg
Post-emesis	PaO₂	253.20%	High	92–98.5%
Post-emesis	A–a Gradient	8.3 mmHg	Normal	5–10 mmHg

Diagnostic imaging and discovery of an ASD

Given the recurrent episodes of unexplained hypoxemia, the patient underwent a stepwise evaluation to investigate for intracardiac or intrapulmonary shunting. An initial TTE with agitated saline contrast (bubble study) was performed. However, the study was technically limited due to suboptimal image quality and an elevated heart rate, which precluded adequate visualization of the interatrial septum. As a result, no definitive shunt could be identified, and the study was deemed nondiagnostic.

To further evaluate for the presence of a right-to-left shunt, a nuclear medicine lung perfusion scan using technetium-99m-labeled macroaggregated albumin (Tc-99m MAA) was conducted. Following intravenous administration of 5.5 mCi of Tc-99m MAA, whole-body scintigraphy was performed. The scan demonstrated homogeneous radiotracer uptake throughout both lungs, with no appreciable extrapulmonary uptake, including the brain. Although the shunt quantification was measured at approximately 8.2%, this was below the 10% threshold typically considered functionally significant and was partially confounded by artifactual activity at the injection site. Overall, the study did not reveal evidence of a functionally significant right-to-left intrapulmonary shunt.

Given the persistence of hypoxemia and clinical suspicion for an intermittent or pressure-dependent shunt, a TEE with bubble contrast was subsequently performed. As the patient was under moderate sedation and unable to perform an effective Valsalva maneuver, manual abdominal compression was utilized to increase intra-abdominal and right atrial (RA) pressures. This maneuver successfully provoked a right-to-left shunt through a 2.6 cm² secundum ASD, which had gone undetected on the previous studies. This dynamic test confirmed the diagnosis and correlated well with the patient’s episodic hypoxemia and prior improvement following abdominal decompression.

**Figure 1:**
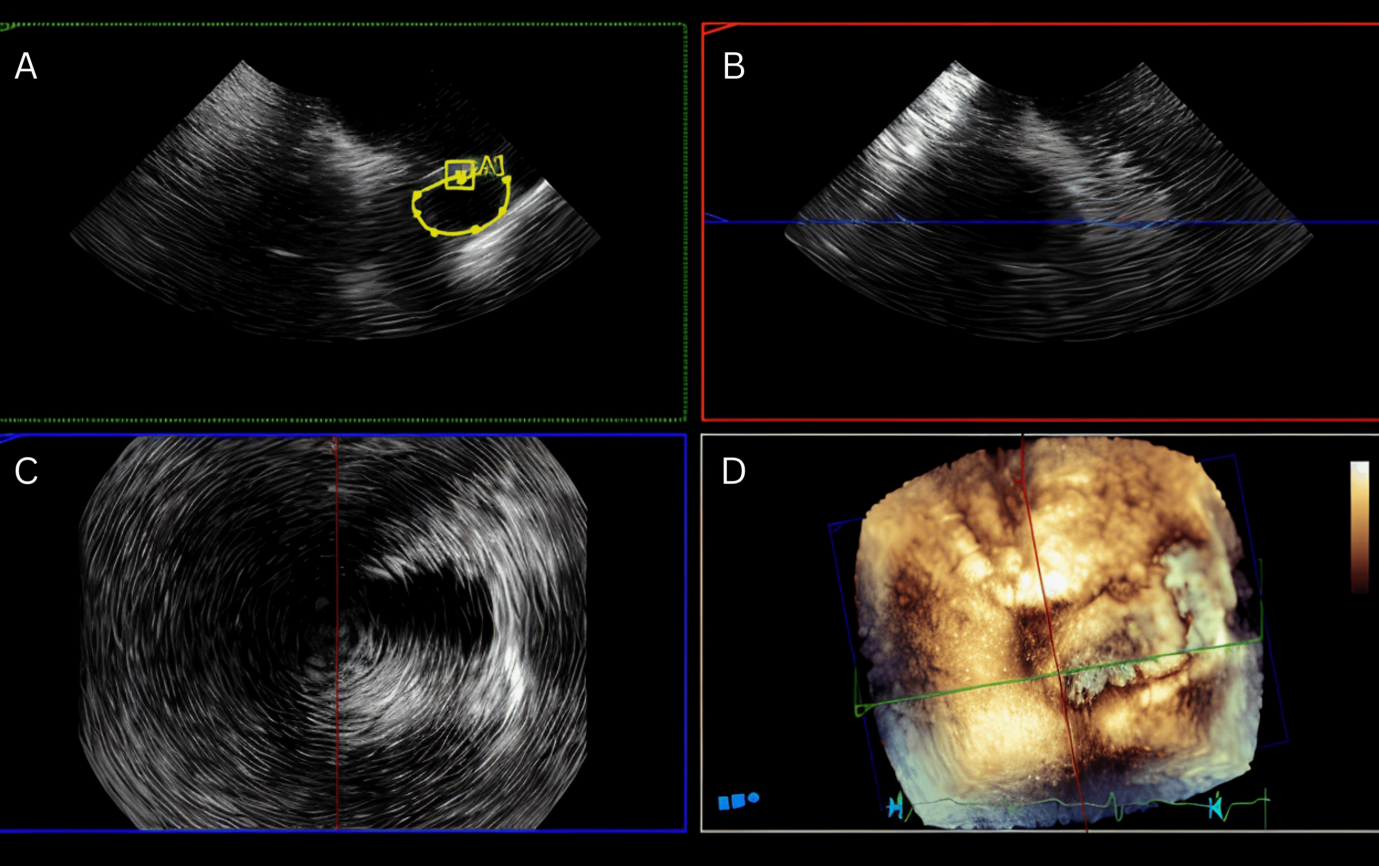
Secundum ASD-TEE views A: A plane of MultiVue on the Philips EPIQ CVx showing a Secundum ASD measuring 2.6cm^2^ (yellow ovoid), B and C: A plane of MultiVue on the Philips EPIQ CVx, D: MultiVue image alignment with 3D rendering visualization of an ASD ASD: atrial septal defect; TEE: transesophageal echocardiography

**Figure 2:**
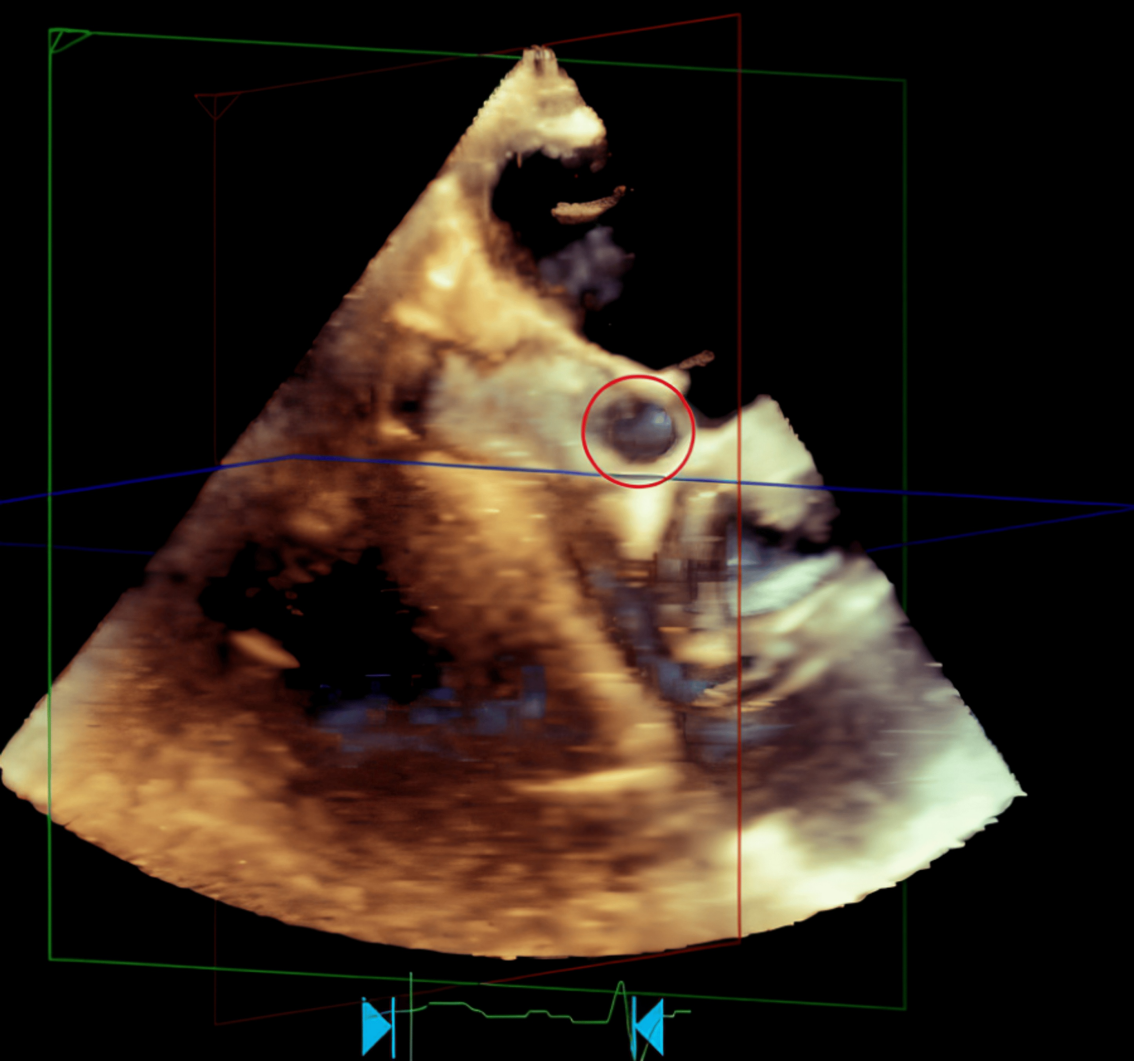
Secundum ASD-TEE 3D view Right atrial en face view showing secundum ASD (red circle) ASD: atrial septal defect; TEE: transesophageal echocardiography

**Figure 3:**
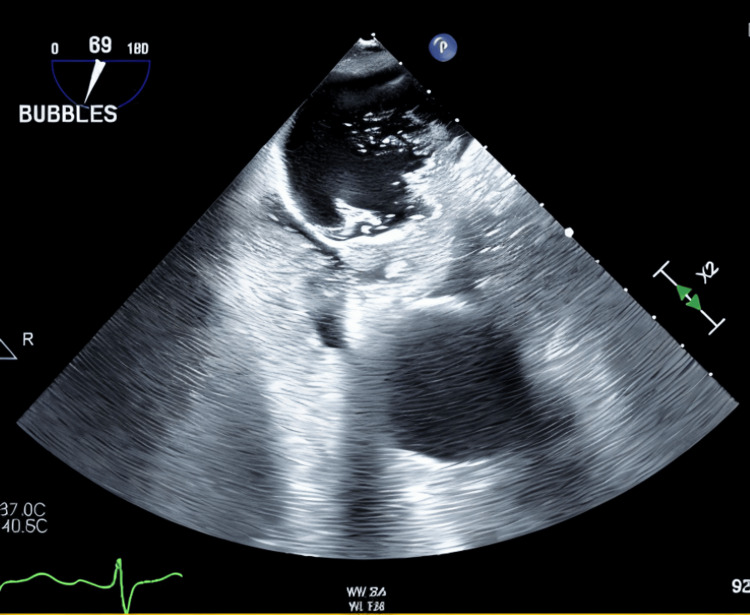
TEE 2D view with bubble study Still frame from agitated saline-enhanced TEE image demonstrating dense opacification of the right atrium with agitated saline contrast. Then microbubbles enter the left atrium almost simultaneously, within one cardiac cycle of right atrial opacification. This is consistent with a right-to-left intracardiac shunt. Findings consistent with an ASD. ASD: atrial septal defect; TEE: transesophageal echocardiography

Figures 1-3 and Video 1 illustrate the anatomical and functional findings of the ASD as demonstrated during the TEE with contrast study.

**Video 1:** A cine loop of TEE with bubble study Agitated saline contrast was visualized entering the right atrium and then microbubbles were seen entering the left atrium almost simultaneously, within one cardiac cycle of right atrial opacification. This is a positive study for right-to-left shunt at the level of the interatrial septum. Findings are consistent with ASD. ASD: atrial septal defect; TEE: transesophageal echocardiography

Cardiac structural and hemodynamic evaluation

TTE revealed RA and right ventricular (RV) dilation, consistent with chronic left-to-right shunting and subsequent right heart volume overload. The RV end-diastolic diameter measured 4.5 cm (normal <4.1 cm), and the RV end-systolic diameter was 3.8 cm, both indicative of dilatation. The RA area was 22 cm² in the apical four-chamber view (normal <18 cm²). Left-sided chambers were within normal limits.

There was mild tricuspid regurgitation with a peak tricuspid regurgitant velocity of 2.8 m/s, corresponding to an estimated RV systolic pressure of 36 mmHg, assuming a RA pressure of 10 mmHg. No pulmonary regurgitation was noted. These findings suggest mild pulmonary pressure elevation but not sufficient to meet the criteria for pulmonary hypertension.

Intervention and outcome

The patient underwent successful percutaneous closure of the ASD using a septal occluder device. Post-procedure, oxygenation remained stable and normoxic without supplemental oxygen. The patient was discharged in good condition and has remained symptom-free on follow-up.

## Discussion

ASDs are among the most common congenital cardiac anomalies diagnosed in adults, accounting for approximately 10% of all such conditions in this population [1]. The secundum subtype, which arises from incomplete fusion or resorption of the septum primum during fetal development, is the most prevalent form [2]. These defects often result in left-to-right shunting and remain asymptomatic. However, under certain hemodynamic conditions, such as elevated RA pressure, intermittent or even persistent right-to-left shunting may occur, leading to paradoxical hypoxemia [3-5]. Triggers for such pressure shifts include pulmonary hypertension, tricuspid regurgitation, or extrathoracic influences such as changes in intrathoracic or intra-abdominal pressure (IAP) [9].

This case underscores the importance of recognizing extrathoracic factors, such as ileus and emesis, that can acutely raise IAP, alter venous return, and precipitate transient right-to-left shunting in patients with undiagnosed or hemodynamically borderline ASDs [10]. In our patient, spontaneous emesis and subsequent nasogastric decompression resulted in the evacuation of over two liters of bilious fluid, coinciding with a dramatic increase in arterial PaO₂ (from 33.3 mmHg to 253.2 mmHg) and normalization of the A-a gradient from 77.2 mmHg to 8.3 mmHg. These changes strongly suggested that the hypoxemia was due to a transient, dynamic intracardiac shunt.

The A-a gradient is a critical physiological metric that helps differentiate causes of hypoxemia, particularly distinguishing between hypoventilation, V/Q mismatch, diffusion defects, and shunting. In this case, the markedly elevated A-a gradient, in the absence of radiographic parenchymal abnormalities, supported the presence of a right-to-left shunt rather than a pulmonary or ventilatory mechanism [11]. Following decompression, the abrupt gradient normalization reinforced the hypothesis of a pressure-dependent shunt. Thus, serial A-a gradient measurements not only identified impaired oxygenation but also guided the clinical team toward advanced diagnostic strategies.

Despite the high clinical suspicion, initial investigations, including TTE with agitated saline contrast and a Tc-99m MAA lung perfusion scan, were nondiagnostic. The TTE was technically limited due to elevated heart rate and suboptimal windows, and the nuclear study showed a shunt fraction of only 8.2%, below the 10% threshold for functional significance [12]. Notably, the nuclear study was also confounded by injection-site artifact, and both tests were likely performed during periods of low RA pressure, conditions under which shunting was unlikely to be observed.

In light of these limitations, the team proceeded to TEE with contrast, during which the patient was sedated and unable to perform a Valsalva maneuver. Instead, manual abdominal compression was employed to simulate increased IAP. This provocative maneuver successfully revealed a 2.6 cm² secundum ASD with dense right-to-left contrast passage, confirming the presence of a significant intracardiac shunt. This diagnostic strategy highlights the utility of provocative maneuvers during TEE, particularly when evaluating for dynamic or intermittent shunts [13].

TCD with agitated saline is another useful adjunctive modality in such cases. Although TCD lacks anatomic specificity, it can noninvasively detect cerebral microbubble signals indicative of paradoxical embolism or shunt physiology and is particularly valuable when TEE is inconclusive or contraindicated [7].

Therapeutically, management should be individualized based on the shunt’s hemodynamic burden and underlying pathophysiology. In the absence of pulmonary hypertension, as in our case, pulmonary vasodilators such as phosphodiesterase-5 inhibitors or endothelin receptor antagonists are not indicated and may be harmful if used indiscriminately [14]. Diuretics may be appropriate in volume-overloaded states, but definitive management for symptomatic or hemodynamically significant ASDs remains percutaneous closure, which was successfully performed in this case [15]. Closure eliminated the shunt, resolved the hypoxia, and restored functional capacity. Surgical repair is reserved for patients in whom device closure is not feasible due to large defects, inadequate septal rims, or concomitant structural cardiac abnormalities [8,16].

## Conclusions

This case illustrates several important clinical principles. First, intermittent right-to-left shunting across an ASD can be triggered by transient increases in intra-abdominal or intrathoracic pressure and may present as unexplained or episodic hypoxemia. Second, standard diagnostic modalities, such as TTE and lung perfusion imaging, can miss pressure- or posture-dependent shunts, particularly when performed under non-provocative conditions. Third, the A-a gradient serves as a powerful tool in identifying shunt physiology and guiding escalation of diagnostic evaluation. Lastly, TEE with provocative maneuvers remains the gold standard for detecting dynamic intracardiac shunts and should be pursued when clinical suspicion persists despite nondiagnostic imaging.

Timely recognition and definitive closure of ASDs can result in the resolution of symptoms, normalization of oxygenation, and prevention of long-term complications, including paradoxical embolism. Maintaining a high index of suspicion and a systematic approach is essential in the evaluation of hypoxia in the absence of overt pulmonary or cardiac pathology.
